# On the Right Track to Treat Movement Disorders: Promising Therapeutic Approaches for Parkinson’s and Huntington’s Disease

**DOI:** 10.3389/fnagi.2020.571185

**Published:** 2020-09-03

**Authors:** Paulina Troncoso-Escudero, Denisse Sepulveda, Rodrigo Pérez-Arancibia, Alejandra V. Parra, Javiera Arcos, Felipe Grunenwald, Rene L. Vidal

**Affiliations:** ^1^Center for Integrative Biology, Faculty of Sciences, Universidad Mayor, Santiago, Chile; ^2^Faculty of Medicine, Biomedical Neuroscience Institute, University of Chile, Santiago, Chile; ^3^Program of Cellular and Molecular Biology, Institute of Biomedical Sciences, University of Chile, Santiago, Chile; ^4^Center for Geroscience, Brain Health, and Metabolism, University of Chile, Santiago, Chile

**Keywords:** Parkinson’s disease, Huntington’s disease, neurotrophic factors, pharmacological therapy, gene modifiers, cellular replacement

## Abstract

Movement disorders are neurological conditions in which patients manifest a diverse range of movement impairments. Distinct structures within the basal ganglia of the brain, an area involved in movement regulation, are differentially affected for every disease. Among the most studied movement disorder conditions are Parkinson’s (PD) and Huntington’s disease (HD), in which the deregulation of the movement circuitry due to the loss of specific neuronal populations in basal ganglia is the underlying cause of motor symptoms. These symptoms are due to the loss principally of dopaminergic neurons of the substantia nigra (SN) par compacta and the GABAergic neurons of the striatum in PD and HD, respectively. Although these diseases were described in the 19th century, no effective treatment can slow down, reverse, or stop disease progression. Available pharmacological therapies have been focused on preventing or alleviating motor symptoms to improve the quality of life of patients, but these drugs are not able to mitigate the progressive neurodegeneration. Currently, considerable therapeutic advances have been achieved seeking a more efficacious and durable therapeutic effect. Here, we will focus on the new advances of several therapeutic approaches for PD and HD, starting with the available pharmacological treatments to alleviate the motor symptoms in both diseases. Then, we describe therapeutic strategies that aim to restore specific neuronal populations or their activity. Among the discussed strategies, the use of Neurotrophic factors (NTFs) and genetic approaches to prevent the neuronal loss in these diseases will be described. We will highlight strategies that have been evaluated in both Parkinson’s and Huntington’s patients, and also the ones with strong preclinical evidence. These current therapeutic techniques represent the most promising tools for the safe treatment of both diseases, specifically those aimed to avoid neuronal loss during disease progression.

## Introduction

**Figure 1:**
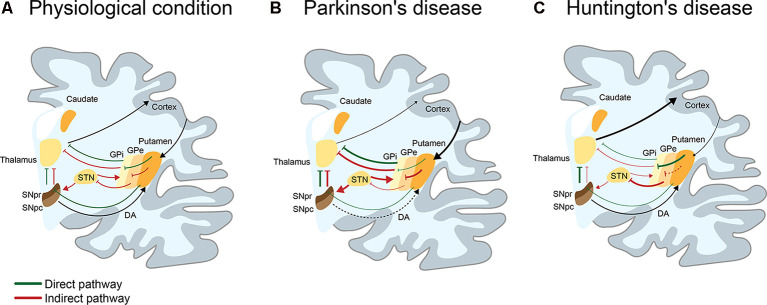
I Schematic representation of the direct and indirect pathways in basal ganglia in physiological, Parkinson’s (PD), and Huntington’s disease (HD) conditions. **(A)** Physiologically, the direct (green line) pathway participates in the activation of movement. This pathway is engaged when the activation of the cortex produces a release of glutamate into the striatum, activating GABAergic medium spiny neurons (MSNs) of the direct pathway. By releasing GABA to the substantia nigra pars reticulata (SNpr) and the internal globus pallidus (GPi), MSNs inhibit neurons of the SNpr/GPi that are also GABAergic. This causes activation of the glutamatergic neurons present in the thalamus, which projects to the cortex, resulting in the activation of movements. On the contrary, the indirect pathway (red line) participates in the inhibition of movement. When GABAergic MSNs that indirectly project to the SNpr through the external globus pallidus (GPe) and the subthalamic nucleus (STN), release GABA into the GPe, inhibits GABAergic neurons present in the GPe. This leads to the disinhibition of the glutamatergic neurons of the STN, which activates GABAergic neurons of the SNpr/GPi. These neurons inhibit neurons present in the thalamus, resulting in a reduction of movement. The selection and execution of movement reflect a dynamic balance between both pathways. **(B)** In PD, the loss of dopaminergic neurons of the SNpc, induces an overactivation of the indirect pathway and decrease of movement. Consequently, there is an increase of GABAergic activity of the GPi/SNpr over thalamic neurons that project to the cortex, leading to loss of movement (hypokinetic disorder). **(C)** In HD (early stage), MSNs of the indirect pathway appear to be affected before the MSNs of the direct pathway. This induces an increase of GABAergic (or inhibitor) activity of the GPe over the STN, which causes the loss of inhibitory activity of the GPi/SNpr over thalamic neurons that project to cortex, leading to the appearance of choreic movements (hyperkinetic disorder).

Movement disorders are characterized by disabilities in speed, fluency, quality, and ease of motor execution, impairments that could be due to an excess or lack of voluntary movements (Shipton, 2012). Movement is produced by the coordinated action of several cortical and subcortical brain structures such as the spinal cord, brainstem, cerebral cortex, cerebellum, and basal ganglia, which collectively fine-tune voluntary and involuntary movements (Ferreira-Pinto et al., 2018). Particularly, the basal ganglia structure comprises a group of subcortical nuclei including the striatum (subdivided in most mammals in caudate and putamen), internal globus pallidus (GPi) and external globus pallidus (GPe), substantia nigra (SN) pars reticulata (SNpr) and compacta (SNpc), and subthalamic nucleus (STN), that together with the primary motor cortex and the thalamus, comprise the motor circuit involved in the control of voluntary movement (Albin et al., 1989; Obeso et al., 2008; Calabresi et al., 2014). The striatum is composed of medium spiny neurons (MSNs), a type of GABAergic neurons representing 90–95% of striatal neurons (Dubé et al., 1988), cholinergic and GABAergic interneurons (Lapper and Bolam, 1992). MSNs are innervated by glutamatergic (excitatory) inputs from the cortex and thalamus, together with dopaminergic inputs from the SN (Pickel et al., 1992). In turn, MSNs differentially express dopamine (DA) D1 or D2 receptors, which determine their participation in two motor circuits: the direct or indirect pathways. The direct pathway is composed of the striatum, GPi, SNpr, thalamus, and motor cortex, promoting the activation of movement through the inhibition of the GPi, in addition to the consequent disinhibition of the thalamus. The indirect pathway, formed by the striatum, GPe, STN, SNpr, thalamus, and motor cortex favors the inhibition of movement by activating the STN with the consequent inhibition of the thalamus (Figure 1A; Calabresi et al., 2014). Alterations in these brain regions are associated with a spectrum of abnormal movement disorders. Among the most studied movement disorders are Parkinson’s (PD) and Huntington’s disease (HD; Figures 1B,C), which are the main focus of this review.

PD is the second most common chronic and progressive neurological disorder with no effective cure (Emamzadeh and Surguchov, 2018) PD incidence is currently around 200 in 100,000 persons (Ball et al., 2019) and the number of PD patients is expected to reach 10 million by 2030 (Dorsey et al., 2007; Ball et al., 2019). PD is mainly an idiopathic disorder with multifactorial etiology, with aging being one of its main risk factors (Hou et al., 2019). Nevertheless, genetic factors have been associated with 5–10% of PD cases (Kim and Alcalay, 2017). Clinical features of PD are a combination of motor symptoms including muscle rigidity, gait difficulty, postural instability, bradykinesia, and tremor at rest (Rees et al., 2018). Also, non-motor symptoms include sensory and sleep alterations, constipation, cognitive impairment, dementia, anxiety, depression, and mood disorders at an early stage of the disease (Munhoz et al., 2015).

One of the two neuropathological criteria required for the diagnosis of PD is the progressive loss of dopaminergic neurons within the SNpc of the basal ganglia (Figure 1B; Agid, 1991; Hirsch et al., 1999). However, not all dopaminergic neurons are equally vulnerable: those that project their axons to the putamen are more vulnerable than those that project their axons to cognitive areas. The second pathological criteria is the presence of α-synuclein (α-syn)-positive inclusions accumulated in Lewy bodies in neurons (Double, 2012). Although the main hallmarks of PD are well described, diagnosis before classic clinical features occur is not currently achievable. Motor symptoms are observed when 70% of the striatal dopaminergic neurons terminals are lost and half of the dopaminergic neurons in the whole brain have degenerated (Double, 2012; Surmeier et al., 2017; Fu et al., 2018).

The current PD treatment is a pharmacological therapy that increases DA levels to provide symptomatic relief. Unfortunately, this therapeutic strategy has limited efficacy. However, new therapeutic alternatives under development attempt to modify the pathology of PD by increasing DA production or improving neuronal health. These approaches efficiently delay neurodegeneration as well as PD symptoms in preclinical models and patients. Currently, finding new treatments that modify pathological PD progression, prevent the dopaminergic neuronal loss, counteract aberrant neuronal activity, and delay the appearance of motor symptoms is the principal goal of many investigations.

HD is the world’s most common monogenic neurological disorder (Huntington’s Disease Collaborative Research Group, 1993), characterized by its autosomal dominant inheritance, midlife onset and progressive course with a combination of motor, cognitive and behavioral features. HD is caused by a mutation in the gene that encodes for the protein huntingtin (HTT), which leads to an expanded CAG trinucleotide, causing an abnormally long polyglutamine (polyQ) tract in HTT. This repetition ranges between 6–35 glutamine units in the normal population. When this tract is ≥40 glutamines long, the mutation is highly penetrant, triggering a disease process that leads to the onset of motor symptoms. Mutant HTT (mHTT) exhibits gain-of-toxic properties, causing dysfunction and death of GABAergic MSNs of the striatum, which is particularly vulnerable to mHTT toxicity (Figure 1C). Once signs and symptoms begin, they progress inexorably throughout the illness, which is inevitably fatal, with a median survival from motor onset of 18 years (Ross et al., 2014).

We describe current pharmacological therapies for both diseases, cellular replacement strategies to restore lost neuronal populations, administration of Neurotrophic factors (NTFs) to increase neuronal viability and health, electrical neuromodulation to restore movement circuitry lost, and genetic approaches to decrease mHTT and α-syn levels. We focused on studies that have reached clinical testing, highlighting preclinical evidence that supports those clinical trials. The clinical trials mentioned throughout this review are summarized in Tables s 1, 2. For those strategies in which clinical trials have not been performed, we present the current state of investigations and remark the important drawbacks that must be solved before these therapeutic approaches jump into clinical trials.

**Table 1:** Parkinson’s disease (PD) therapies under clinical trials.

Sponsor	CT Identifier	Stage	Administration	Target	Description	Start–completion date
**Cellular replacement**
University of Cambridge	NCT01898390	Phase1. Active, not recruiting	Transplant	Striatum	TRANSEURO Open Label Transplant Study in Parkinson’s disease (TRANSEURO)	May 2012–March 2021
Chinese Academy of Sciences	NCT03119636	Phase 1–2. Recruiting	Transplant (stereotaxis)	Striatum	Safety and Efficacy Study of Human ESC-derived Neural Precursor Cells in the Treatment of Parkinson’s disease	May 2017–December 2021
Hebei Newtherapy Bio-Pharma technology Company Limited	NCT03550183	Phase 1. Recruiting	Intravenous infusion	Not specified	Umbilical Cord Derived Mesenchymal Stem Cells Therapy in Parkinson’s disease	January 2018–December 2022
Bundang CHA Hospital	NCT01860794	Phase 1–2. Recruiting	Transplant	Striatum	Evaluation of Safety and Tolerability of Fetal Mesencephalic Dopamine Neuronal Precursor Cells for Parkinson’s disease	May 2012–April 2022
**Growth factors administration**
North Bristol NHS Trust	NCT03652363	Phase 2. Completed	Bilateral Intraputamenal Infusions of GDNF Administered *via* Convection enhanced delivery	Striatum	GDNF in ideopathic Parkinson’s disease	October 2012–April 2016
Herantis Pharma Plc.	NCT03295786	Phase 1–2. Complete	Intraputamenal DDS	Striatum	CDNF brain infusion in Parkinson’s disease patients	September 2017–January 2020
Herantis Pharma Plc.	NCT03775538	Phase 1–2. Active, not recruiting	Intraputamenal DDS	Striatum	Safety study of CDNF brain infusion in Parkinson’s disease patients	December 2018–January 2020
Newron Sweden AB	NCT02408562	Phase 1–2. Complete	Intracerebroventricular	Not specified	Safety and tolerability study of rhPDGF-BB in Parkinson’s disease patients	April 2015–January 2016
Newron Sweden AB	NCT01807338	Phase 1–2. Complete	Intracerebroventricular	Not specified	PDGF-BB in Parkinson’s disease patients	March 2013–October 2014
**Electrical stimulation**
Northwell Health	NCT04184791	Phase No aplica. Recruiting	DBS	STN	Study the effect of protocol a 60 HZ of STN-DBS in Parkinson’s patients with gait disorder	January 2020–December 2021
University of Miami	NCT02022735	Phase No aplica. Active, recruiting yet	DBS	Not specified	Evaluation of different parameters of stimulation for the treatment of gait disorder in Parkinson’s patient	December 2013–December 2025
Boston Scientific Corporation	NCT01221948	Phase 2. Completed.	DBS	STN	Evaluation of effectiviness and safe of Boston scientific implantable DBS Vercise system for treatment of moderate to severe idiopathic Parkinson’s disease	October 2010–June 2018
University of Minnesota	NCT00053625	Phase 3. Completed	DBS	STN & GPi	Evaluation of STN-DBS and GPi-DBS in neuropsychological and psychiatric function	February 2003–April 2015
University of Minnesota	NCT02709148	Phase 1. Active, not recruiting	DBS	STN & GPi	Study of relation between brain activity in Parkinson’s disease patients and DBS, by recording of LFP in cortex	July 2017–July 2020
Western University, Canada	NCT03079310	Phase Not applicable. Recruiting	SCS	Spinal cord	Thoracic Dorsal Spinal Cord Stimulation for the Treatment of Gait and Balance Impairments in Parkinson’s disease	February 2016–April 2020
**Gene therapy approaches**
Oxford BioMedica	NCT00627588	Phase 1–2. Complete	Stereotactic injection of ProSavin	Striatum	Safety and efficacy of ProSavin for treatment of idopathic Parkinson’s disease patients	March 2008–May 2013
Axovant Sciences Limited	NCT01856439	Phase 1–2. Active, not recruiting	Stereotactic injection of ProSavin	Striatum	Long Term study of ProSavin in Parkinson’s disease	May 2013–July 2019

**Table 2:** Huntington’s disease therapies under clinical trials.

Sponsor	CT Identifier	Stage	Administration	Target	Description	Start–completion date
**Electrical stimulation**
Heinrich-Heine University, Duesseldorf	NCT02535884	Phase Not Applicable. Recruiting	DBS	GP	Study of efficacy and safety of pallidal DBS in HD patients to improve motor function. Device: ACTIVA^®^ PC neurostimulator (Model 37601)	July 2014–October 2020
**DNA targeting approaches**
Ionis Pharmaceuticals, Inc.	NCT02519036	Phase 1–2. Complete	Intrathecal	CNS	Safety, Tolerability, Pharmacokinetics, and Pharmacodynamics of ISIS 443139 in Participants With Early Manifest Huntington’s Disease	August 2015–May 2019
Hoffmann-La Roche	NCT03342053	Phase 2. Complete	Intrathecal	CNS	This study will test the safety, tolerability, pharmacokinetics and pharmacodynamics of RO7234292 administered intrathecally to adult patients with Huntington’s Disease	November 2017–June 2020
Hoffmann-La Roche	NCT03761849	Phase 3. Recruiting	Intrathecal	CNS	A Study to Evaluate the Efficacy and Safety of Intrathecally Administered RO7234292 (RG6042) in Patients With Manifest Huntington’s Disease	January 2019–September 2022
Wave Life Sciences Limited	NCT03225833	Phase 1b–2a. Recruiting	Intrathecal	CNS	Safety and Tolerability of WVE-120101 in Patients With Huntington’s Disease (PRECISION-HD1)	July 2017–December 2020
Wave Life Sciences Limited	NCT03225846	Phase 1b–2a. Recruiting	Intrathecal	CNS	Safety and Tolerability of WVE-120102 in Patients With Huntington’s Disease (PRECISION-HD2)	July 2017–December 2020
**RNA targeting approaches**
UniQure Biopharma B.V.	NCT04120493	Phase 1–2. Recruiting	Stereotaxic	Striatum	Safety and Proof-of-Concept (POC) Study With AMT-130 in Adults With Early Manifest Huntington Disease	September 2019–May 2026

## Current Pharmacological Treatments to Alleviate Motor Symptoms in PD and HD

Currently, no approved drugs can modify the progression of either PD or HD. Available pharmacological therapies only aim to treat motor symptoms to improve the quality of life of patients but, ultimately, these drugs do not mitigate the progressive neurodegeneration.

For PD, pharmacological approaches are designed to reestablish DA levels through: (i) increasing DA availability using DA precursors like levodopa (L-dopa) or dopaminergic agonists like pramipexole, and (ii) inhibiting DA degradation by monoamine oxidase B inhibitors [MAO-BI, like selegiline (Eldepryl^®^) and rasagiline (Azilect^®^)] or catechol-O-methyl transferase inhibitors [COMTI, like entacapone (Comtan^®^)] and tolcapone (Tasmar^®^; Van de Schyf, 2015; Teijido and Cacabelos, 2018; Carrera and Cacabelos, 2019). A caveat in the chronic administration of anti-parkinsonian drugs is the *“wearing-off”* phenomenon, which produces additional psychomotor and autonomic complications, like levodopa-induced dyskinesia (LID; Fahn et al., 2004; Stacy, 2009; Ammal Kaidery et al., 2013; Cacabelos, 2017). Moreover, L-dopa pharmacokinetics is unpredictable and commonly leads to administration increase, complex regimens, and poor patient compliance. Nevertheless, L-dopa remains as the gold standard pharmacological intervention for motor symptoms in PD patients (Oertel and Schulz, 2016; Fox et al., 2018). Although the pathophysiology of *wearing-off* and dyskinesia is complex and not completely understood, it appears to be linked to the short plasma half-life of L-dopa, as short-acting dopaminergic drugs can induce alterations in brain DA concentrations which lead to motor dysfunction (Olanow et al., 2006; Rajan et al., 2017). To assess this problem, strategies that prolong L-dopa plasma half-life have been developed, including the administration of COMTI. A significant quantity of orally administered L-dopa is metabolized to 3-O-methyldopa (a useless metabolite) by COMT in the gastrointestinal tract. By inhibiting COMT, more L-dopa will be absorbed, increasing its bioavailability and extending its half-life (Oertel and Schulz, 2016). Some of these drugs are tolcapone, entacapone, and opicapone (Parkinson Study Group, 1997; Rinne et al., 1998; Poewe et al., 2002; Cacabelos, 2017). However, the FDA has restricted the use of tolcapone due to hepatic necrosis leading to death (Haasio et al., 2001). Common combinations include L-dopa/carbidopa (an L-dopa decarboxylation inhibitor; Sinemet^®^) and L-dopa/benserazide (Madopar^®^), looking to prevent systemic adverse effects related to the peripheral metabolism of L-dopa to DA, including nausea, dyskinesia, motor fluctuations, hypotension, psychiatric symptoms (especially hallucinations) and diaphoresis. Duodopa, an intestinal gel form of L-dopa/carbidopa, provides a more stable response to L-dopa. Stalevo^®^ is a triple-drug, containing carbidopa, L-dopa, and entacapone (Rezak, 2007). Another strategy for prolonging L-dopa half-life is the inhibition of MAO-B, the breakdown-enzyme of DA in the brain, increasing DA levels at synapses. Selegiline, through its metabolite desmethylselegiline, has also shown anti-apoptotic effects (Tatton et al., 1996; Parkinson Study Group, 2005; Cacabelos, 2017). Rasagiline is a significantly more potent MAO-BI that reduces the motor symptoms in PD patients and has disease-modifying potentials (Parkinson Study Group, 2005; Rascol et al., 2005; Olanow et al., 2009). IPX066 (Rytary) is an improved oral formulation of L-dopa approved in 2015 by the FDA containing both an immediate-release and a sustained-release L-dopa. Published phase II and III studies show that IPX066 improved Unified Parkinson’s Disease Rating Scale (UPDRS) motor scores compared to placebo in early PD patients and reduced *wearing-off* in advanced PD patients (Kestenbaum and Fahn, 2015). Before L-dopa, anticholinergics were the treatment of choice for PD. However, MAO-BI and modern DA agonists have largely replaced these drugs. Trihexyphenidyl (Artane^®^), benztropine (Cogentin^®^), and procyclidine (Kemadrin^®^) is usually reserved for tremor resistant to dopaminergic agents. The adverse effects of anticholinergics include blurred vision, dry mucus membranes, urinary retention, and cognitive changes (Rezak, 2007).

Dopamine receptor (DR) agonists mimic DA actions in the brain by directly stimulating DRs. Two common DR agonists are pramipexole (Mirapex^®^) and ropinirole (Requip^®^). These directly stimulate post-synaptic D2 and D3 receptors in the striatum and are prescribed as monotherapy or combined with L-dopa. Potential side effects of pramipexole and ropinirole include hypotension, sleep, cognitive/psychiatric alterations, dyskinesias, and compulsive behavior. The latter is believed to be a result of DA dysregulation in the limbic and frontal circuits that are connected to the basal ganglia (Evans and Lees, 2004). Polyoxazoline (POZ) polymer conjugation for continuous dopaminergic drug delivery may improve motor symptoms while avoiding side effects. The *in vitro* and *in vivo* pharmacokinetics of POZ-conjugated rotigotine (DA agonist) was characterized, demonstrating that the sustained dopaminergic stimulation profile achieved by POZ-conjugated rotigotine formulations, could represent a significant advance in the treatment of PD. POZ polymer administration can improve motor symptoms in a rat model of PD (Eskow Jaunarajs et al., 2013; Fox et al., 2018). For HD, the only approved pharmacological therapy for the treatment of motor symptoms is tetrabenazine, a vesicular monoamine transporter 2 (VMAT-2) inhibitor that reduces DA neurotransmission *via* its depletion from presynaptic vesicles, resulting in a reduction of chorea manifested by HD patients (Wyant et al., 2017). However, its side effects, which include sedation, anxiety, depression, and suicidality have limited its use (Wyant et al., 2017; Dean and Sung, 2018). In 2017, the FDA approved a deuterated derivative of tetrabenazine, deutetrabenazine, with improved pharmacokinetic profile, allowing a less frequent daily dosage with comparable systemic exposure of the drug, resulting in less adverse events (Dean and Sung, 2018). No other small molecules are currently used for HD treatment, but several therapeutic approaches are in clinical trials, which will be discussed later.

## Cellular Replacement Therapies for PD and HD

**Figure 2:**
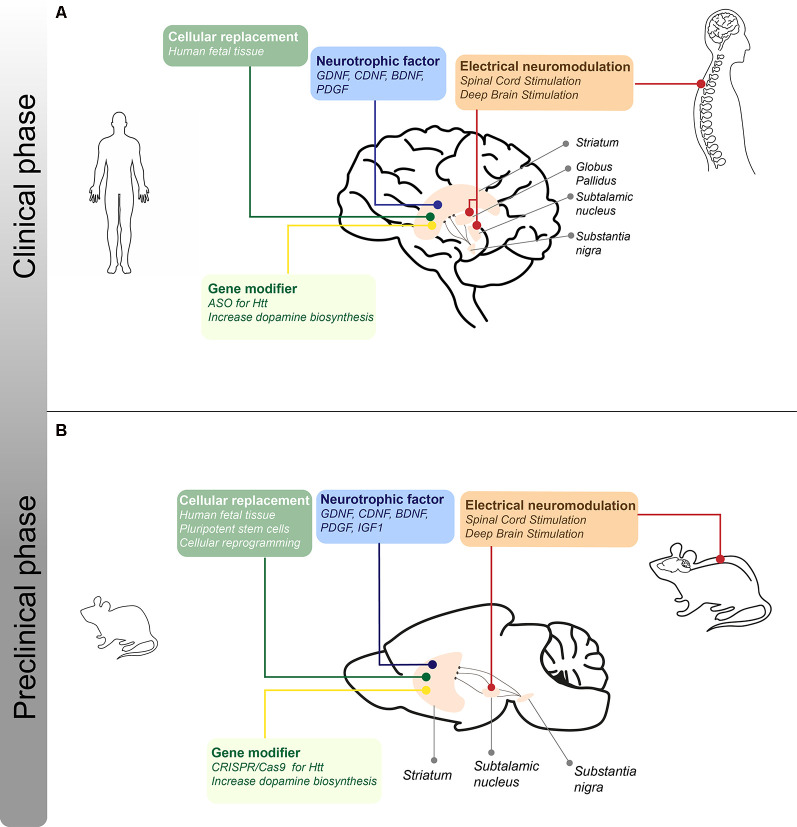
I Schematic representation of the different treatments for motor symptoms for PD and HD under clinical and preclinical phases. **(A)** Approaches regarding cellular, neurotrophic, electrical, and gene-modifying therapies that are in clinical trials or stage of optimization for PD and HD patients. The lines indicate the different brain structures that are the target of the therapeutic strategies mentioned. **(B)** Approaches regarding cellular, neurotrophic, electrical, and disease-modifying therapies in the preclinical phase using rodent models of PD and/or HD. The lines indicate the different brain structures that are the target of the therapeutic strategies mentioned.

In 1967, in an important breakthrough, Cotzias et al. (1967) demonstrated that the administration of a precursor of DA, L-dopa, improved motor function in PD patients, leading to the thought that the cure for PD was discovered. Also in the 1960s, tetrabenazine was introduced as an antipsychotic but also showed beneficial effects for the treatment of hyperkinetic motor symptoms, like chorea in HD patients (Dalby, 1969; Huntington Study Group, 2006). To date, it is known that these drugs do not reverse disease progression and in many cases do not have the desired effects. This has brought the idea that local production of DA and GABA, and therefore the replacement of the neurons that produce it, would be the ideal treatment for these diseases. The fact that the major symptoms present in PD and HD patients are due to the loss of dopaminergic and GABAergic neurons in specific brain regions, respectively, means that replacing these specific cell types could help relieve some of the symptoms present in patients. This has given rise to different branches of investigations seeking cellular replacement-based therapies, which have shown promising results in animal models for these diseases as well as in affected patients (Figure 2).

### Human Fetal Tissue as a Source of Progenitor Cells

The first study demonstrating that dopaminergic neurons could be replaced using fetal tissue was performed using 6-hydroxydopamine (6-OHDA)-lesioned rats that were implanted with DA-rich ventral mesencephalic tissue from rat fetuses (Björklund and Stenevi, 1979; Perlow et al., 1979). These studies were followed by the generation of the first non-human primates PD model: monkeys lesioned with 1-methyl-4-phenyl-1,2,3,6-tetrahydropyridine (MPTP; Burns et al., 1983). This model manifested several of the patient’s symptoms, and transplanting primate fetal mesencephalic tissue into their striatum showed to alleviate these symptoms (Bakay et al., 1985; Sladek et al., 1987; Taylor et al., 1991). These studies set foot for the first PD cell replacement therapy in humans. These clinical trials were performed using dopaminergic neuron precursors from human fetal tissue, which were transplanted into the striatum of PD patients (Lindvall et al., 1989, 1992; Freed et al., 1990, 1992). Transplanted tissue presented no negative effects at the transplantation site, was functional and survived in the transplanted brain region, but clinical benefits were variable (Freed et al., 1992; Kordower et al., 1998; Hagell et al., 1999; Li et al., 2016).

It is important to understand that dopaminergic neurons engrafted in the striatum are deprived of their SNpc afferents. Instead, when dopaminergic neurons are transplanted in the striatum, they may form connections with cortical, intrastriatal, and thalamic neurons, which are not normally connected with nigral dopaminergic neurons. Moreover, considering the nature of fetal tissue, possibly other types of neurons and glial cells differentiate in the grafted brain area. Additionally, only a portion of the grafted fetal tissue corresponds to cells that needed restoration in the specific brain region. Another important concern is the presence of α-syn aggregates and Lewy body inclusions in the grafted cells in PD patients (Kordower et al., 2008; Li et al., 2008). This highlights the necessity of using a complementary strategy that allows donor cells to be resistant to the spreading of α-syn.

On the other hand, by the mid-1980s, the first studies using fetal tissue were performed in rat models of HD (Table 4). These studies demonstrated that the grafted tissue survived, was functional and recovered some of the behavioral alterations present in HD rats (Deckel et al., 1983; Isacson et al., 1984, 1985; Sanberg et al., 1986). The *in vivo* functionality of the grafted striatal fetal tissue was also assessed, showing GABA release upon dopaminergic and glutamatergic inputs (Campbell et al., 1993). Diverse studies further demonstrated the effect of striatal fetal tissue transplantation in diverse pharmacological models of HD (Hantraye et al., 1992; Nakao et al., 1999) and by late 1990s, the first clinical trials in HD patients were performed. HD patients were injected unilaterally or bilaterally with fetal tissue, which was originated from various donated embryos. Given the amount of tissue needed and their origin from different embryos, this strategy causes immune rejection by the patient’s immune system. Therefore, complementary immunosuppression therapy is needed, which has shown no adverse effects on patients (Freeman et al., 2000). After fetal cell transplantation, patients presented improved cognitive function and stabilization of motor functions, which worsen a few years after the surgery (Kopyov et al., 1998; Gallina et al., 2008; also see Table 1).

**Table 3:** Common animal models of Parkinson’s Disease.

Model	Characteristics	Reference
**1. Mouse models**
**Pharmacological models**
6-OHDA	Stereotaxic injection in medial forebrain bundle	Zigmond and Stricker (1984)
MPTP	IP injection induce loss of dopaminergic neurons of nigrostriatal pathway	Sonsalla et al. (1987) and Sonsalla and Heikkila (1988)
Rotenone	Stereotaxic injection in parenchyma caused damage in dopaminergic nigrostriatal pathway	Heikkila et al. (1985)
Reserpine	Impairment in monoamines storage in intracellular vesicles disrupting motor activity	Spina and Cohen (1989) and Cooper et al. (2004)
**Genetically modified models**
A53T	Mutation leads formation of neuronal inclusions leading neurodegeneration	Giasson et al. (2002)
PINK1 transgenic mice (knockout)	Display impaired dopamine release, but not dopaminergic neurons degeneration	Kitada et al. (2007)
Parkin transgenic mice (knockout)	Display abnormalities in dopamine transmission, but not dopaminergic neuron degeneration	Perez et al. (2005)
DJ-1 transgenic mice (knockout)	Display abnormalities in dopamine transmission, but not dopaminergic neuron degeneration	Goldberg et al. (2005)
LRKK2 transgenic mice (overexpression)	Display dopaminergic dysfunction and some behavioral deficits, but not dopaminergic neurons degeneration	Lin et al. (2009)
**Recombinant adeno-associated viral vector (AVV) models**
Human WT-α-synuclein	Direct injection in SN induce progressive loss of dopaminergic neuron	St. Martin et al. (2007)
Human-A53T-α-synuclein	Direct injection in SN induce progressive loss of dopaminergic neuron	Oliveras-Salvá et al. (2013)
**2. Rat models**
**Pharmacological models**
6-OHDA	Direct administration in the brain (striatum, subtantia nigra or median forebrain bundle) cause the loss of dopaminergic neurons	Ungerstedt (1968)
Haloperidol	IP injection block striatal dopamine transmission	Sanberg (1980)
Rotenone	IV or IP administration cause nigrostriatal dopaminergic degeneration	Betarbet et al. (2000)
**Recombinant adeno-associated viral vector (AVV) models**
Human WT-α-synuclein Human-A53T-α-synuclein	Direct injection in SN induce progressive loss of dopaminergic neurons and motor impairtment	Kirik et al. (2002a,b)
Human A30P-α-synuclein	Direct injection in SN induce progressive loss of dopaminergic neurons and motor impairtment	Klein et al. (2002)
**3. Large models**
**Pharmacological models**
MPTP rhesus monkey	IP injection induce loss of dopaminergic neurons of nigrostriatal pathway	Burns et al. (1983)
MPTP squirrel monkey	IP injection induce loss of dopaminergic neurons of nigrostriatal pathway	Langston et al. (1984)
MPTP marmoset monkey	IP injection induce loss of dopaminergic neurons of nigrostriatal pathway	Jenner et al. (1984)

**Table 4:** Common animal models of Huntington’s disease.

Model	Characteristics	Reference
**1. Mouse models**
**Genetically modified models**
R6/2	Expresses human exon 1 of HTT with ~150 glutamine repeats	Mangiarini et al. (1996)
R6/1	Expresses human exon 1 of HTT with ~115 glutamine repeats	Mangiarini et al. (1996) and Naver et al. (2003)
N171-82Q	Expresses a 171 amino acid mutant HTT fragment with 82 glutamine repeats	Schilling et al. (1999)
YAC128	Expresses full length human HTT with 128 glutamine repeats	Slow et al. (2003) and Van Raamsdonk et al. (2005)
BACHD	Expresess full lenght human HTT with 97 glutamine repeats	Gray et al. (2008)
HdhQ111	knock-in mouse having human *HTT* exon 1 sequence with 111 glutamine repeats	Wheeler et al. (2000)
HdhQ140	knock-in mouse having human *HTT* exon 1 sequence with 140 glutamine repeats	Menalled et al. (2003)
HdhQ150	knock-in mouse having mouse *HTT* exon 1 sequence with 150 glutamine repeats	Lin et al. (2001)
**Pharmacological models**		
Quinolonic acid (QA)	QA directly administered to the striatum induces striatal neurodegeneration	McLin et al. (2006)
3-Nitropropionic acid (3-NP)	Repeated injections of 3-NP produce excitotoxic-like lesions of the striatum	Gould et al. (1985)
**2. Rat models**		
Pharmacological models		
Quinolonic acid (QA)	QA directly administered to the striatum induces striatal neurodegeneration	Bordelon et al. (1997)
3-Nitropropionic acid (3-NP)	Repeated injections of 3-NP produce excitotoxic-like lesions of the striatum	Gould et al. (1985)
Kainic acid	Intrastriatal injection produces selective degeneration of neurons	Coyle et al. (1978)
Ibotenic acid (IBO)	Central microinjections induce lesions in the striatum	Smith et al. (1987)
**3. Larger animal models**		
Pharmacological models		
HD rhesus monkey	Expresses mutant exon 1 HTT with 84 glutamine repeats	Yang et al. (2008)
HD pigs (N208-105Q)	Expresses 208 N-terminal aminoacids of mutant HTT with 105 glutamine repeats	Yang et al. (2010)

Although several clinical trials have been performed using fetal tissue transplants to treat PD and HD patients, this technique has a few but important limitations. First, is important to consider that when using human fetal tissue, tumors can develop, which could be explained by the presence of actively dividing immature neuroepithelial cells (Keene et al., 2009). Second, not only neuronal loss must be corrected, but also the loss of glial cells. Astrocytes are the most abundant cell type found in the brain (Miller, 2018) and play important roles in maintaining brain homeostasis, supporting a neuronal activity, and metabolism. In PD and HD, there is a deregulation of astrocyte activity, including electrophysiological changes, calcium homeostasis, glutamate reuptake, and metabolism, among others (Booth et al., 2017; Garcia et al., 2019; Gray, 2019). Therefore, the replacement of the lost neurons should be accompanied by a replacement or modification of the glial cells that support them.

### Pluripotent Stem Cells as a Source of Differentiated Cell

Pluripotent stem cells (PSCs) are an unlimited source of cells with the potential to give rise to any type of cell of the body. Cells differentiated from embryonic stem cells (ESCs) and induced pluripotent stem cells (iPSCs; Takahashi and Yamanaka, 2006) are widely used as *in vitro* models for many diseases, including neurodegenerative diseases, and also as a source of cell-replacement therapies. Initial studies demonstrated that, when midbrain-derived dopaminergic neurons where grafted in the striatum of rodent models of PD (Table 3), long-term survival of these cells was observed, which were tyrosine hydroxylase (TH)-positive neurons, completely reversed amphetamine-induced rotational behavior and lacked neuronal overgrowth (Kriks et al., 2011). Importantly, midbrain human dopaminergic neurons grafted in MPTP-lesioned non-human primates survived in the grafted area, expressed TH, extended fibers to the surrounding striatum, and did not present neuronal overgrowth (Kriks et al., 2011).

However, one limitation regarding the use of PSCs-derived neurons is the greater immune reaction observed with allogeneic grafts compared with isogenic cells (Duan et al., 1995; Morizane et al., 2013). Using both approaches, Hallett et al. (2015) demonstrated that in a non-human primate PD model autologous iPSCs-derived midbrain-like dopaminergic neurons could successfully engraft and survive for as long as 2 years. This led to improving motor function and complete re-innervation in the striatum with extensive axonal outgrowth, and no graft overgrowth, tumor formation or inflammation was observed (Hallett et al., 2015). Also, as clinical trials are underway, establishing the optimal and safest protocol for dopaminergic neurons differentiation is necessary to obtain the adequate number of neurons that permit improvements in patient’s motor symptoms. Also, the characterization of the graft is relevant, as the differentiation protocol could give rise to other cell types that may alter the physiological conditions in the grafted site. One important concern about the use of autologous transplantation using iPSC-derived dopaminergic neurons of PD patients is that these cells will carry any intracellular dysfunction related to disease pathogenesis. It is important to remember that dopaminergic neuron replacement is primarily focused on the treatment of motor symptoms. Patients in which symptoms like dementia or other cognitive impairment are also present may not be benefited completely with this type of therapy.

One key issue for cellular transplantation into human brains is the necessity of an important amount of cells. Fetal tissue cells not only provide a limited number of cells but also come with ethical and religious concerns. Therefore, the use of iPSCs obtained from somatic cells of patients is excellent for personalized cell-based therapy and to model HD *in vitro*. The first research group to obtain striatal neurons from HD iPSCs-derived neuronal stem cells (NSCs) was Zhang et al. (2010), who used iPSCs derived from an HD patient (Park et al., 2008). These cells not only express MSNs markers but also could be used as an excellent model for drug screening in HD research (Zhang et al., 2010). Using the same HD iPSCs, Zhang et al. (2010) demonstrated that these cells can be corrected for the CAG mutation by replacing the expanded 72 CAG repeat with a normal 20–21 CAG repeat (An et al., 2012). Finally, the corrected iPSCs-derived NSCs could be successfully differentiated into MSNs *in vitro*, and when transplanted into the striatum of R6/2 mice (Table 4) they differentiated into MSNs neurons (An et al., 2012). The genetic modification of human iPSCs not only brings us closer to the proper modeling of diseases but also provides a potential therapy. It has been demonstrated that iPSCs-derived neuronal cells from an HD preclinical model develop cellular features of HD cells, which could be rescued by genetic suppression of HTT and pharmacological treatment (Carter et al., 2014). Using human ESCs (hESCs) or human iPSCs (hiPSCs) differentiated into MSNs progenitors, it has been demonstrated that the transplantation of these cells into the striatum of rodent HD models can form functional connections with other cells, and project their axons to other structures involved in the movement circuitry, like the SN (Faedo et al., 2017; Adil et al., 2018).

As highlighted previously, since HD is caused by a genetic mutation, and differentiated MSNs progenitors come from HD patients, it is imperative to correct the mutation present in these cells, along with the replacement of the target neurons and other cell types, like interneurons and glial cells, as they may provide a healthy and functional environment for the new neurons to integrate to the local circuitry and survive. Currently, no clinical trials are assessing the use of PSCs in HD patients.

### Cellular Reprogramming for PD and HD

In the adult brain, NSCs are present in the subventricular zone of the lateral ventricle and the subgranular zone of the dentate gyrus. These NSCs are capable of generating neuroblasts, which differentiate into mature neurons (Zhao et al., 2008; Ma et al., 2009). Despite the presence of a niche for the generation of new neurons, these cells have limited migration to remote regions, like the SN and striatum. Hence, the idea to generate new local neurons from preexisting cells has been studied for the last 10 years. Initial studies have demonstrated that fibroblasts can be reprogrammed to dopaminergic neurons through the ectopic expression of transcription factors (Caiazzo et al., 2011; Kim et al., 2011; Pfisterer et al., 2011). Considering the reprogramming of cells for the treatment of neurodegenerative diseases, astrocytes were initially considered as an attractive alternative and their reprogramming to neurons forming functional synapses was demonstrated (Berninger et al., 2007; Heinrich et al., 2010).

The first studies in which dopaminergic neurons were generated by reprogramming human and mouse fibroblasts using lineage-specific factors for the conversion to dopaminergic neurons (Caiazzo et al., 2011; Kim et al., 2011). The converted neurons were positive for several dopaminergic markers and expressed genes related to the dopaminergic lineage rather than with the fibroblast of origin (Caiazzo et al., 2011; Kim et al., 2011). Converted dopaminergic neurons formed synapses, had synaptic activity in culture, and showed electrophysiological properties similar to dopaminergic neurons (Caiazzo et al., 2011). The transplantation of these cells into the brain of wild type (Caiazzo et al., 2011) and PD mice (Kim et al., 2011) showed that converted neurons integrated with the host tissue expressed dopaminergic markers and had electrophysiological responses, which led to an improvement in mice behavior (Kim et al., 2011).

Also, Addis et al. (2011) were the first to demonstrate that astrocytes could be reprogrammed into dopaminergic neurons *in vitro*. The obtained neurons displayed an up-regulation of genes expressed by dopaminergic neurons, along with electrophysiological properties, including the spontaneous firing of action potentials observed in dopaminergic neurons (Addis et al., 2011). A few years later, the first human astrocytes were directly converted into functionally competent dopaminergic neurons *in vitro* (Rivetti di Val Cervo et al., 2017) and it was described for the first time the *in vivo* conversion of astrocytes into dopaminergic neurons in the 6-OHDA mouse model of PD (Rivetti di Val Cervo et al., 2017). These converted dopaminergic neurons were excitable and expressed dopaminergic neuron markers, which helped relieve the cycling behavior observed in these animals. These and other reports have shown that the cellular reprogramming relies on the expression of lineage-specific transcription factors. However, it has been shown that downregulation of PTB in mouse and human fibroblasts, an RNA-binding protein negatively controlling neuronal induction and maturation, induces the conversion of these cells into functional neurons (Xue et al., 2013, 2016). Considering these finding and that downregulation of PTB occurs during neurogenesis (Hu et al., 2018), Qian et al. (2020) recently demonstrated using the 6-OHDA mouse model of PD that adeno-associated virus (AAV)-mediated downregulation of PTB using an shRNA convert nigral astrocytes into functional dopaminergic neurons. These converted neurons integrate into the nigrostriatal pathway, extending their axons into the striatum and other brain regions. These neurons were electrophysiologically functional and restored the striatal dopamine lost due to the 6-OHDA treatment, leading to a reversal of the motor deficits observed in these mice (Qian et al., 2020). Importantly, these results were also observed using an antisense oligonucleotide against PTB, giving these findings a potentially clinical approach for the treatment of PD in patients (Qian et al., 2020).

Not only glial cells can originate dopaminergic neurons. In an elegant work performed by Niu et al. (2018), authors demonstrated that striatal neurons could be reprogramed to dopaminergic-like neurons in the adult mouse striatum. These neurons, although expressing both dopaminergic and GABAergic markers, have electrophysiological properties like endogenous dopaminergic but no MSNs neurons. These dopaminergic-like neurons were also functionally connected with surrounding neurons, confirmed by the presence of spontaneous postsynaptic currents (Niu et al., 2018). Hence, these results seem to be promising for converting MSNs into dopaminergic neurons under pathological conditions.

Initial studies have shown that striatal astrocytes can be reprogramed into proliferative neuroblasts in young, adult and aged mice brains (Niu et al., 2013), which are interesting especially for HD, a disease of adult-onset. Furthermore, when these neuroblasts were treated with NTFs or histone deacetylase inhibitor, they differentiated into mature neurons with electrophysiological properties (Niu et al., 2013). Using (AAV)-based conversion, striatal GABAergic, and glutamatergic neurons could be originated after reprogramming NG2 glial cells (Torper et al., 2015). Newly generated neurons presented electrophysiological properties of functional neurons, remained stable for a long period, and even integrated into local neuronal circuitry (Torper et al., 2015). Consequently, the use of endogenous glial cells for the regeneration of neuronal population lost under neurodegenerative conditions has emerged as an interesting source, avoiding the use of differentiated external cells and therefore minimizing the possible immunorejection of foreign cells (Li and Chen, 2016; Srivastava and DeWitt, 2016; Barker et al., 2018).

Recently, using AAV-based reprogramming of striatal astrocytes, Wu et al. (2020) demonstrated that astrocytes could be converted to MSNs in the striatum of R6/2 and YAC128 mice. Converted neurons expressed specific MSNs markers, showed electrophysiological properties, and projected their axonal terminals to the GP and SNpr. All these findings were accompanied by a reduction in striatal atrophy, attenuation of the phenotypic deficit, and an extended life span of R6/2 mice with converted MSNs (Wu et al., 2020).

Nevertheless, it is important to complement these reprogram therapies with a therapy that targets the mutation in the HTT gene. Converted neurons will sooner or later express and accumulate mHTT, which will eventually lead to neurodegeneration. Therefore, *in vivo* reprogramming of glial cells into healthy MSNs has an important clinical potential, which must also be combined with gene therapy strategies to reduce or ablate mHTT expression in these new neurons. Also, the application of this approach in the clinic is challenging by the lack of standardized protocols for cellular reprogramming, as well as the efficiency of converted cells. This depends on the donor cell, the type of cell that is needed, the characteristics of patients that must be considered, i.e., age, the severity of the disease, and treatment with other drugs, among others. Although challenging, *in vivo* cell reprogramming appears as the most promising therapy candidate for cellular replacement for PD and HD patients.

## Neurotrophic Factors-Based Therapies for PD and HD

NTFs are molecules that promote the differentiation, myelination, and survival of neurons, which are also involved in the neuroinflammatory response (Fernandez and Torres-Aleman, 2012; Labandeira-Garcia et al., 2017). A reduction in the bioavailability of NTFs in the peripheral and central system during aging suggests a role of these factors during neurodegenerative disorders such as PD and HD (Zuccato and Cattaneo, 2007; Gasperi and Castellano, 2010; Procaccini et al., 2016; Salem et al., 2016). NTFs, like Glial cell-line Derived Neurotrophic Factor (GDNF), Brain-Derived Neurotrophic Factor (BDNF), Cerebral Dopamine Neurotrophic Factor (CDNF), Mesencephalic astrocyte-derived Neurotrophic Factor (MANF), Platelet-Derived Growth Factor (PDGF), Insulin-like Growth Factors (IGFs), and others have been through preclinical and clinical trials for PD and HD (Figure 2). Here, we describe the recently therapeutic approaches based on the restoration of NTFs levels in the brain to prevent and/or stop the neurodegenerative process describe in PD and HD.

### Glial-Derived Neurotrophic Factor (GDNF)

GDNF is considered as a neuro-restorative therapeutic protein that induces the regeneration of dopaminergic neurons given that enhances dopaminergic cell survival and differentiation *in vitro* (Lin et al., 1993; Zurn et al., 2001). Besides, GDNF has shown a protective effect on the survival of noradrenergic neurons in the locus coeruleus (Arenas et al., 1995), an affected region in neurodegenerative diseases such as PD and HD (Zweig et al., 1992; Oertel and Schulz, 2016). The neuroprotective effects of GDNF have prompted preclinical and clinical studies. Chronic infusion of GDNF into the lateral ventricle or the striatum promoted the restoration of the nigrostriatal dopaminergic system and significantly improved motor functions in a rhesus monkey PD model (Grondin et al., 2002). Moreover, GDNF protects nigral dopaminergic neurons from degeneration and improves motor behavior in 6-OHDA rat models of PD (Tereshchenko et al., 2014). However, GDNF has limited use due to its inability to cross the blood-brain barrier (BBB), therefore new administration methods have been explored, including: (1) the delivery of GDNF in biodegradable microspheres (Garbayo et al., 2009); (2) gene therapy using DNA nanoparticle (DNP) technology for the expression of human GDNF (hGDNF) in the striatum (Fletcher et al., 2011); (3) the use of intra-cerebroventricular (ICV) catheters implanted into the basal ganglia (Gill et al., 2003; Nutt et al., 2003); (4) the use of viral vectors (Kordower et al., 2000); and (5) GDNF-producing fibroblasts (Grandoso et al., 2007). ICV administration of recombinant hGDNF to non-human primates showed to significantly improve locomotor activity after 4 months of treatment (Zhang et al., 1997). Also, intraputamenal (Ipu) delivery of GDNF in MPTP-lesioned non-human primates significantly increased DA release (Grondin et al., 2003). Despite the positive results in the survival of dopaminergic neurons and improvements in motor behavior (Gill et al., 2003; Patel et al., 2005; Lang et al., 2006), the invasiveness of the delivery of GDNF to the brain represents a limitation for its use.

The first attempt to probe the benefits of GDNF in PD patients consisted of the ICV administration through catheter implantation in 50 PD patients for 8 months. Patients presented side effects after drug administration, mainly weight loss, nausea, and vomiting. At the end of treatment, patients did not present improvements in the UPDRS motor scores (Nutt et al., 2003). Similarly, delivery through Ipu infusion of recombinant hGDNF in 34 PD patients did not observe significant improvements in UPDRS motor scores (Lang et al., 2006). Furthermore, in a completed clinical study with 42 PD patients, bilateral Ipu GDNF infusions every 4 weeks for 9 months showed that ^18^F-DOPA analyzed through PET scan imaging had a significant increase in the putamen, but no significant changes in UPDRS scores were registered. However, the extended treatment for 18 months showed significant improvements in the UPDRS motor scores (Whone A. et al., 2019; Whone A. L. et al., 2019). Additionally, a phase I study showed an important improvement in UPDRS motor scores after 1 year of GDNF Ipu therapy (Slevin et al., 2005, 2007). Completed clinical studies have demonstrated the safety and potential efficacy of Ipu GDNF infusion, with no evidence of GDNF-induced toxicity (Slevin et al., 2005). However, antibodies were detected in some patients and device-related problems were reported (Lang et al., 2006; Slevin et al., 2007). It has been shown that the effect of GDNF *in vitro* and *in vivo* requires TGF-β (Peterziel et al., 2002). The combined effect of GDNF-TGFβ showed a strong neuroprotective effect in rodent PD models (Peterziel et al., 2002) and future therapies may include the simultaneous use of both molecules. Finding a non-invasive and safe way to deliver GDNF is key to evaluate this NTF as an effective treatment for PD.

Preclinical studies in rat models of HD have demonstrated the benefits of ICV injection of GDNF in restoring the excitotoxic-induced damage in the striatum, amelioration of amphetamine-induced rotational behavior (Araujo and Hilt, 1997) and locomotor activity improvement (Araujo and Hilt, 1998). However, no registered clinical trials are testing the efficacy of GDNF in HD patients.

### Cerebral Dopamine Neurotrophic Factor (CDNF) and Mesencephalic Astrocyte-Derived Neurotrophic Factor (MANF)

In 2003, a protein called mesencephalic astrocyte-derived neurotrophic factor (MANF) was characterized and demonstrated to promote survival of embryonic dopaminergic neurons *in vitro* (Petrova et al., 2003). Then, a homologous protein called CDNF was discovered with a protective role for dopaminergic neurons. Several studies evidence the protective role of CDNF and MANF in dopaminergic neurons against the injury caused by α-syn oligomers (Latge et al., 2015). The intrastriatal injection of CDNF prevents the loss of TH-positive neurons in a 6-OHDA-lesioned rat model of PD (Lindholm et al., 2007), and protected dopaminergic neurons in 6-OHDA and MPTP mouse models of PD (Lindholm et al., 2007; Voutilainen et al., 2009). MANF has been tested in the 6-OHDA-lesioned rat model showing beneficial effects (Voutilainen et al., 2009). CDNF and MANF diffuse to the brain significantly better than GDNF, and CDNF was more efficient in reducing amphetamine-induced ipsilateral rotations in the 6-OHDA rat PD model in comparison with GDNF treatment (Voutilainen et al., 2011). In 6-OHDA-lesioned monkeys, PET imaging showed a significant increase of DA transporter (DAT) ligand-binding activity in lesioned animals treated with CDNF (Garea-Rodríguez et al., 2016).

The first phase I–II clinical trial using CDNF in PD patients is being conducted since 2017. In this study, an implanted drug delivery system (DDS) for Ipu of recombinant human CDNF is used in patients with idiopathic mild-advanced PD (Table 1, NCT03295786). Additionally, another phase I–II clinical trial to evaluate the beneficial effects of CDNF in PD patients is still on course (Table 1, NCT03775538). Currently, the delivery of CDNF for HD treatment has not been described.

### Brain-Derived Neurotrophic Factor (BDNF)

BDNF is the most abundant NTFs in the brain (Barde et al., 1982), mostly involved in physiological processes including morphological and functional synaptic plasticity, long-term potentiation, learning and memory (Bramham and Messaoudi, 2005; Lu et al., 2014). In the CNS, BDNF binds specifically to tropomyosin-related kinase receptors B (TrkB) receptors and its signaling cascade is involved in neuronal survival (Kaplan and Miller, 2000). Interestingly, MAOI (i.e., rasagiline and selegiline) prevent dopaminergic neuron loss by increasing BDNF expression and other NTFs (Weinreb et al., 2007; Maruyama and Naoi, 2013).

Studies have demonstrated that a decrease of BDNF is implicated in neurological disorders (Siegel and Chauhan, 2000; Takahashi et al., 2012; Lu et al., 2013). Thus, strategies for developing quantification and modulation of BDNF levels represent a viable approach for biomarker and treatment development, respectively, being useful for a variety of neurodegenerative diseases (Lu et al., 2013; Song et al., 2015). Post-mortem studies reveal that BDNF is significantly reduced in nigrostriatal dopaminergic neurons from PD patients (Mogi et al., 1999; Parain et al., 1999; Howells et al., 2000). A decrease of BDNF in serum from PD patients has also been observed (Wang et al., 2016). Also, BDNF offers neuroprotection of striatal neurons, and supporting studies have shown that BDNF levels are decreased in the brains of HD rodent models (Conforti et al., 2008) and patients (Ferrer et al., 2000; Zuccato and Cattaneo, 2007).

A study with 42 HD patients revealed that BDNF serum concentrations were significantly lower in patients compared to healthy controls (Ciammola et al., 2007). However, a later study analyzed 398 blood samples, indicating that mRNA and protein levels of BDNF between HD and healthy controls were not significantly different, questioning its potential as a biomarker for early diagnosis of HD (Zuccato et al., 2011).

Although the contribution of BDNF on PD and HD pathology is robust, no clinical trials are currently testing its safety and efficacy for the treatment of these diseases.

### Platelet-Derived Growth Factor (PDGF)

Classic studies indicate that PDGF has diverse functions in organs, including the stimulation of cell proliferation (Heldin and Westermark, 1999). Different isoforms of PDGF can be found in tissues, in which the PDGF-BB isoform has shown a protective effect in cultured dopaminergic neurons (Pietz et al., 1996). After the treatment of rats with 6-OHDA, PDGF-BB was increased, suggesting a compensatory response (Funa et al., 1996) and PDGF-BB injections induced functional recovery and provided neuroprotection of the nigrostriatal system in a PD mouse model (Zachrisson et al., 2011). PDGF-BB might be acting on neural progenitors and stem cells in the subventricular zone, promoting neurogenesis (Zachrisson et al., 2011). A study with 12 PD patients demonstrated that the administration of PDGF-BB into the brain ventricles for 2 weeks was well tolerated with no evident or aggressive side effects, and an increase in DAT binding was noted in the putamen of PDGF-BB-treated patients (Paul et al., 2015). Considering that PDGF-BB can stimulate neurogenesis, it may be possible to evaluate the co-treatment with PDGF-BB and other NTFs or drugs to restore the nigrostriatal pathway and promote neuroprotection in PD.

Both *in* vitro (Nakao et al., 1994) and *in vivo* reports (Sjöborg et al., 1998) have related the effect of PDGF in HD. PDGF-BB exerts trophic effects in developing rat DARPP32-positive striatal neurons in culture, suggesting the possibility that PDGF-BB might participate in the development and maintenance of striatal neurons *in vivo*, and could be used to modulate the neurodegeneration in HD models (Nakao et al., 1994). Then, the same group published the expression profile of PDGF in a rat model of HD, generated by unilateral intrastriatal ibotenic acid injections (Table 4). The evidence showed the accumulation of PDGF in astrocytes, suggesting a role of PDGF in a repair process in neurodegeneration (Sjöborg et al., 1998). Currently, no PDGF-based clinical trials are in course for HD treatment.

### Insulin-Like Growth Factor Family (IGFs)

The IGF system is composed of insulin, IGF1, and IGF2, and its receptors: IR, IGF1R, and IGF2R, respectively (Cohen et al., 1991). The use of IGFs as therapy might represent a novel tool for the treatment of neurodegenerative disorders (Ebert et al., 2008). Studies *in vitro* and in preclinical models have demonstrated the neuroprotective effects of IGFs (Jarvis et al., 2007). Administration of IGF1 after injury reduced neuronal loss against several stressors such as oxidative stress, excitotoxicity, hypoxia, hypoglycemia, among others (Suh et al., 2013). Several studies using *in vivo* models of PD demonstrated beneficial effects of IGF1 treatment by preventing dopaminergic neuronal loss in the SN (Ebert et al., 2008), improving motor performance in a rat model of PD (Guan et al., 2000; Krishnamurthi et al., 2004).

IGFs have received interest due to its role in preventing and rescuing striatal neuronal damage which is observed in HD (Lewitt and Boyd, 2019). Increased IGF1 plasma levels were observed in the YAC128 mouse model of HD (Pouladi et al., 2010). Similarly, high IGF1 plasma levels were observed in HD patients and this was associated with cognitive impairment characteristic in this disorder (Saleh et al., 2010), however, the correlation between elevated IGF1 plasma levels and the motor and cognitive impairment in HD remains to be elucidated. Despite the high peripheral levels of IGF1, only small amounts of IGF1 cross the BBB into the brain. In this context, Lopes et al. (2014) showed that intranasal administration of IGF1 significantly improve motor function and restores metabolic changes in YAC128 mice model of HD, demonstrating that intranasal administration allows for the direct delivery of IGF1 into the CNS through the olfactory pathway.

In a recently published study, it has been demonstrated that the AAV administration of IGF2 into the striatum of YAC128 and R6/2 mice decreased the levels of mHTT and increased the levels of DARPP-32, a marker used to assess striatal neurons survival (García-Huerta et al., 2020). Interestingly, neuroprotective effects of IGF2 treatment have been described in preclinical models of others neurodegenerative diseases such as amyotrophic lateral sclerosis (Allodi et al., 2016), spinal muscular atrophy (Brown et al., 2009) and Alzheimer’s disease (Pascual-Lucas et al., 2014). However, today no clinical trials are studying the safety and efficacy of IGF2 as a possible treatment for PD or HD.

## Electrical Neuromodulation Therapies for PD and HD

The cardinal motor symptoms of PD and chorea in HD are caused by the progressive degeneration of dopaminergic neurons in the SNpc (Figure 1B) and the loss of MSNs in the striatum (Figure 1C), respectively. In both cases, the motor impairment is attributed to alteration of functional connectivity of the striatum, a principal input of the basal ganglia (Albin et al., 1989).

Hyperkinetic movement disorders are characterized by uncontrollable and excessive motor activity, as chorea in HD. Reports published between 1987 and 1989 showed that blocking the activity of the STN produces hyperkinetic motor symptoms. Similar results are observed when the GABAergic inputs from the striatum are blocked, favoring the inhibitory (GABAergic) modulation of the GPe over the STN (Crossman, 1987; Crossman et al., 1988; Robertson et al., 1989). On the other hand, hypokinetic disorders like akinesia and bradykinesia have been described in PD. In this case, the decrease in striatal DA levels, as a result of the decrease in its synthesis, release, and reuptake (Lundblad et al., 2012) alter the corticostriatal balance causing an increase in the activity of the indirect pathway and reducing the activity of the direct pathway, that leads to a breakdown of the internal balance of the basal ganglia, and consequently the loss of movement control (Obeso et al., 2008; Galvan et al., 2015). These symptoms, unlike hyperkinetic movements, are treated with DA agonists as L-dopa (Cotzias et al., 1967, 1969). However, as stated in previous sections, the chronic use of this pharmacological therapy has a limited effect, which in the case of PD can induce a motor complication known as LID and on-off phenomenon (Fahn et al., 2004; Stacy, 2009; Ammal Kaidery et al., 2013; Cacabelos, 2017).

Among the therapeutic alternatives proposed in recent decades, electrical neuromodulation therapies, i.e., Deep Brain Stimulation (DBS) and Spinal Cord Stimulation (SCS) have emerged as interesting options for treatment of neurodegenerative pathologies associated with movement disorders such as PD and HD (Figure 2). The effect of stimulation on different basal ganglia nuclei in patients is described below.

### Deep Brain Stimulation (DBS)

DBS is a neurosurgical strategy based on the implantation of electrodes on subcortical nuclei that, through electrical signals, can modulate the neuronal activity of different regions of the brain. The use of DBS for the treatment of motor disorders was first proposed in 1971 by Natalia Bekhtereva (Bekhtereva et al., 1972; Hariz et al., 2010). But it was until 1996 when the FDA approved the stimulation of the ventral intermediate nucleus (VIM) of the thalamus for the treatment of essential tremor and severe tremor in PD. Later in 2002, the stimulation of the STN and GPi for the treatment of bradykinesia and rigidity in advanced cases of PD was included (Strotzer et al., 2019). It has been suggested that the stimulation of the STN induce the suppression of aberrant oscillatory synchronization at low frequency (13–35 Hz, beta band) and this contributes to motor amelioration, mainly bradykinesia and rigidity. However, more studies are necessary to understand the mechanisms involved. Currently, a group at the University of Minnesota is performing a clinical trial to investigate how the brain activity in PD patients is related to DBS and pharmacological management, by the chronic recording of local field potential (LFP) in the cortex (Table 1, NCT02709148).

The underlying molecular and cellular mechanisms of DBS are still not sufficiently identified and different assumptions about functional principles have been proposed (Jakobs et al., 2019). Studies in neurotoxin pre-clinical models of PD (Table 3) have suggested that STN-DBS can induce neuroprotective effects by reducing the loss of dopaminergic neurons (Maesawa et al., 2004; Temel et al., 2006; Wallace et al., 2007; Harnack et al., 2008; Spieles-Engemann et al., 2010; Wu et al., 2012). Additionally, it has been described that STN-DBS induces an increase in the striatal expression of BDNF (Spieles-Engemann et al., 2011), which is described as a powerful anti-apoptotic factor that favors the maintenance and survival of the nigral dopaminergic population (Zhao et al., 2017). As stated before, BDNF would exert its effect by signaling downstream of the TrkB receptor (Fischer et al., 2017). Nevertheless, reports in a genetic model of PD have revealed contradictory results. Musacchio et al. (2017) have reported that DBS reduces the loss of dopaminergic neurons, but it did not rescue the DA deficit. On the other side, Fischer et al. (2017) revealed that STN-DBS cannot counteract the axonopathy and dopaminergic loss progression induced by α-syn overexpression, but the differences in the starting time of application protocol must be considered. Moreover, the application of STN-DBS in pre-clinical models of PD has shown both metabolic and physiological effects on the nigrostriatal DA system, inducing an increase in the levels of striatal DA and its metabolites (Bruet et al., 2001; Meissner et al., 2003; He et al., 2014). Genetic screening has revealed that high frequency-DBS can induce a differential expression of genes involved in apoptosis, growth, and neuroplasticity (Lortet et al., 2013). Studies also revealed that DBS increased the activity of the enzyme TH (Meissner et al., 2003) and the expression of the D1 receptor (Carcenac et al., 2015).

For this reason, different ongoing clinical trials are assessing the long-term neurological, neurophysiological, and neuropsychological effects of DBS treatment (Table 1, NCT00053625, NCT03021031), as well as improving the administration of the treatment (Table 1, NCT03021031). The technical difficulty of STN-DBS in PD patients is that a case-by-case parameter adjustment is necessary. Therefore, different studies are looking for electrophysiology biomarkers that can predict and improve the effectiveness of DBS therapy, as well as new stimulation methodologies (directional DBS system) by invasive and non-invasive recording (Table 1, NCT03353688). Another limitation of DBS is the incapacity to alleviate the freezing of gait (FoG), a symptom observed in more than half of PD patients. Using electroencephalography (EEG) it is crucial to determine electrophysiological biomarkers present in FoG episodes that can be modulated by GPi-DBS or pedunculopontine nucleus stimulation (PPN-DBS, Table 1, NCT02548897). Furthermore, studies are evaluating different parameters of STN-DBS protocols to improve gait disorders (Table 1, NCT04184791, NCT02022735).

DBS of the GP is a promising alternative target for the treatment of chorea, motor symptoms classic in the early stages of HD (Albin et al., 1990). DBS has been considered an alternative to pallidotomy, a neurosurgical strategy for the treatment of choreic movement published in 1952, showing good effects in four HD patients (di Cianni et al., 1992). In 2004, Moro and colleagues (Moro et al., 2004) reported the first case of a 43-year-old patient with an 8-year history of HD submitted to bilateral GP stimulation. They found that stimulation at 40 Hz and 130 Hz improved motor symptoms, reducing chorea and dystonia, although the high frequency worsened bradykinesia. The improvement in the patient’s performance of a motor task was associated with an increase of the activation of the sensorimotor cortex, premotor cortex, supplementary motor area, and anterior cingulate cortex, although high frequency did not modulate the activity in premotor cortex (Moro et al., 2004). Moreover, the simultaneous stimulation of the STN and GPi has shown beneficial effects (Gruber et al., 2014), however, if the simultaneous stimulation has an additive effect is not clear. To date, two prospective, randomized, and double blind studies have been posted. One published in 2015 (Wojtecki et al., 2015), and others still on course (Table 2, NCT02535884). In the first study, six patients, four with chorea-dominant and two with Westphal-variant (rigid-hypokinetic syndrome, often associated with juvenile-onset of HD), underwent DBS treatment for 6 months and showed a reduction of chorea (Wojtecki et al., 2015). Nevertheless, the Westphal-variant patients did not show any improvements, suggesting that GP-DBS cannot ameliorate bradykinesia.

There is no doubt that optimizing DBS systems is a great challenge. Furthermore, understanding the correlation between abnormal brain activity and motor symptoms is necessary to obtain major beneficial results. For a more detailed review of this topic, we suggest some reviews published elsewhere (Anderson and Lenz, 2006; Miocinovic et al., 2013; Fasano and Lozano, 2015).

### Spinal Cord Stimulation (SCS)

Generally used for the treatment of chronic pain since 1967 (Shealy et al., 1967; Dones and Levi, 2018), SCS consists of the application of electrical pulses in the dorsal columns of the spinal cord (directly in the epidural space). It has been suggested that the mechanism of action is based on the antidromic activation of the dorsal column fibers, which activate the inhibitory interneurons within the dorsal horn (Yampolsky et al., 2012). However, the exact mechanisms are not fully elucidated.

Currently, the use of this therapeutic strategy has gone beyond nociceptive control. In 2009, Fuentes and colleagues (Fuentes et al., 2009) proposed the use of SCS for the treatment of motor symptoms in PD. Like this report, in the last decade several studies have evaluated SCS effects in advanced cases of PD, showing interesting effects in axial symptoms (gait and postural dysfunction) both in preclinical (Santana et al., 2014) and clinical studies. These benefits are not observed with other treatments like DBS or L-dopa. The mechanism involved in the effects of SCS in motor symptoms of PD is not fully understood. It has been proposed that SCS, by releasing biphasic electrical pulses of high frequencies (300 Hz), would increase locomotor activity mainly through modulation of activity in the cortex and basal ganglia (Fuentes et al., 2009; Santana et al., 2014). This modulation would occur by activating the path of the dorsal columns, which in turn would modulate the activity of the thalamus, and from there to the cortex and striatum, causing the breakdown of aberrant low-frequency oscillations [beta (10–30 Hz)] observed in preclinical models (Fuentes et al., 2009; Santana et al., 2014) and PD patients (Kuhn et al., 2006), similarly as described for DBS treatment. Nevertheless, this does not explain the effect of SCS on gait and postural dysfunction. The participation of the brainstem, specifically the pedunculopontine nucleus (PPN), has been suggested, given its connection with different structures involved in the motor system, summarized by Chambers et al. (2019).

SCS treatment application methodologies differ among reports. The position of the electrodes varies between patients depending on the study. Electrodes can be placed at the cervical or thoracic level (Cai et al., 2020), showing that in general, both strategies ameliorate motor impairments. The first studies assessing SCS as a treatment for motor symptoms involved PD patients with chronic pain as a primary indication. In most cases, SCS reduced the pain and improved motor function, which could reflect a synergistic effect (Thevathasan et al., 2010; Fénelon et al., 2012; Hassan et al., 2013; Nishioka and Nakajima, 2015; Kobayashi et al., 2018; Mazzone et al., 2019). Recently, Samotus et al. (2018) showed the effectiveness of SCS (300–400 μs/30–130 Hz) in the treatment of FoG and gait in five patients without pain for 6 months. This evidence is encouraging for the search of treatments for symptoms that, to date, have not been satisfactorily controlled. This is the case for gait dysfunctions that affect nearly 40–60% of PD patients and are not improved by dopaminergic therapy (Giladi et al., 2001). Additionally, Pinto de Souza et al. (2017) reported the use and effectiveness of SCS as a complementary strategy to rescue the loss of efficacy of DBS and DA medication. But other studies are currently in a course to evaluate the efficacy of SCS in gait disorders (Table 1, NCT03079310).

To date, three studies in preclinical models have suggested that SCS could counteract the progression of PD through a neuroprotective effect. Shinko et al. (2014) showed in rats that SCS applied regularly for 2 weeks (one session of 1 h daily) starting 2 days before 6-OHDA injection, decreases dopaminergic neuronal loss by 20–25%, and increase the expression of VEGF, which could favor neuroprotection given its ability to reduce dopaminergic neuronal death by suppressing apoptosis (Yasuhara et al., 2004). Similarly, Yadav et al. (2014) demonstrated that the application of an SCS protocol for 6 weeks (two sessions of 30 min per week) that started a week post-6-OHDA injection reduced the loss of dopaminergic neurons by 10%. Furthermore, both studies showed a positive effect in preserving the dopaminergic innervation of the striatum, reducing its loss by 30–35%. Recently, an investigation showed that continuous application of SCS (24 h ON SCS), starting immediately after injection of 6-OHDA, reduced the loss of dopaminergic neurons and their striatal projections by ~35% and ~32% respectively, while a regular SCS protocol (8 h SCS ON/16 h SVS OFF) counteracted the degeneration of the nigrostriatal dopaminergic pathway only by ~23% (Kuwahara et al., 2020). Thus, the periodicity of the treatment application reflects on different degrees of neuroprotection (Kuwahara et al., 2020). These data suggest that SCS could have a neuroprotective effect that might contribute to the relief of the observed motor symptoms in PD, given the relevance of dopaminergic projections in modulating the functioning of the circuit of the nuclei of the base through the regulation of cortical and subcortical neuronal activity during movement (Gerfen and Surmeier, 2011; Canessa et al., 2016). Nevertheless, additional studies are necessary to understand the mechanism involved in the beneficial effects associated with SCS in the long-term. More detailed reviews on this topic can be found elsewhere (de Andrade et al., 2016; Cai et al., 2020).

For HD, SCS treatment has not been suggested, probably because SCS has better effects in alleviating gait and posture problems, while DBS is still the best option for the treatment of involuntary movements, such as chorea.

## Gene Therapies for PD and HD

For the development of new therapies for PD and HD, it is important to include, especially for HD and genetic forms of PD, genetic correction/editing of the mutated gene(s). Nowadays, there are several gene silencing/editing technologies, including RNA interference (RNAi), antisense oligonucleotides (ASO), and clustered interspaced short palindromic repeats (CRISPR/cas9), which can be used as therapies for the treatment of PD and HD. For a more in-depth knowledge of gene therapy delivery systems and other cellular targets, reviews are published elsewhere (Sudhakar and Richardson, 2019; Chen et al., 2020).

As previously stated, PD is characterized by the selective degeneration of dopaminergic neurons in the SN, thus approaches aiming to revert this loss based on the delivery of genes encoding for enzymes required for DA synthesis could be useful. The first enzyme for DA synthesis is TH, which requires the enzyme GTP-cyclohydrolase-1 (GCH-1) to synthesize a cofactor for DA biosynthesis (Daubner et al., 2011). TH converts tyrosine into L-dopa, which finally is converted into DA by the aromatic L-amino acid decarboxylase (AADC; Hadjiconstantinou and Neff, 2008). Therapies to deliver enzymes involved in DA synthesis have been proved in preclinical and clinical studies showing its benefits.

Initially, gene therapy was based on the delivery of separate AAV vectors to transfer two or three enzymes critical for DA biosynthesis. These strategies showed behavioral benefits in rat and non-human primate PD models (Kirik et al., 2002a; Muramatsu et al., 2002). Furthermore, a clinical study in patients with moderate to advanced PD demonstrated the safety and tolerability of a 6 months treatment with a bilateral Ipu of AAV vector encoding for the human AADC gene (AAV-hAADC; Eberling et al., 2008). Importantly, PET scans using an AADC tracer demonstrated an increase in gene expression throughout the study. Similarly, administrating the AAV-hAADC vector in the putamen of PD patients showed to be safe and tolerable (Muramatsu et al., 2010). This study demonstrated the efficacies of AAV vectors for gene delivery, which persisted up to 2 years with a 46% improvement of the UPDRS motor scores (Muramatsu et al., 2010). Another completed study showed that bilateral Ipu infusion of the AAV-hAADC vector improves UPDRS mean scores by 30% in PD patients after 6 months (Table 1, NCT00229736). However, this study reported 3 cases in which surgical procedures caused intracranial hemorrhage, showing an important limitation of the surgical procedure, but not necessarily of the therapeutic strategy of gene delivery (Christine et al., 2009). Consequently, a long-term evaluation study of AAV-hAADC demonstrated stable transgene expression over 4 years after vector delivery in PD patients (Mittermeyer et al., 2012).

To potentiate the effects and benefits of the enzyme therapy for DA synthesis, researchers have developed a strategy using a simple vector, which carries the genes that encode for the three key enzymes for DA biosynthesis. One of these strategies uses a lentiviral-based vector derived from the equine infectious anemia virus (EIAV; Azzouz et al., 2002). This tricistronic lentivirus vector encodes TH, AADC and CH1 (Lenti-TH-AADC-CH1) in a single vector (ProSavin). Delivery of Lenti-TH-AADC-CH1 vector into the striatum of a non-human primate PD model restored extracellular concentrations of DA and improved motor function for up to 12 months (Jarraya et al., 2009). Furthermore, ProSavin was administered into the striatum of PD patients, demonstrating a significant improvement in mean UPDRS motor scores at 6 months post-treatment, with no adverse effects detected (Table 1, NCT00627588). Additionally, a long-term study of ProSavin showed that the treatment was well-tolerated and safe, but its clinical benefits are still under observation after 4 years (Palfi et al., 2014).

Other alternatives using gene delivery for PD treatment are focused on lowering α-syn levels in dopaminergic neurons. One study used a ribozyme combined with an AAV vector (rAAV-SynRz). Nigrostriatal injection of rAAV-SynRz in MPP+-treated adult rats, which has increased expression of α-syn (Kühn et al., 2003), resulted in down-regulation of α-syn, preventing its accumulation in the SN, and significantly protected TH-positive neurons (Hayashita-Kinoh et al., 2006). Despite these positive results, no clinical trials using gene therapy for silencing α-syn to treat PD are being conducted.

One important branch of investigation regarding HD therapies is the reduction of HTT DNA, RNA, and/or protein levels. Using a conditional transgenic mouse model of HD, it has been demonstrated that the reduction of mHTT ameliorates motor and psychiatric-like deficits, forebrain weight loss, cortical and striatal volume decrease, presynaptic and postsynaptic markers changes, and electrophysiological changes in these mice (Wang et al., 2014). One possible alternative to reduce mHTT levels is the use of DNA-targeting strategies (Ambrose et al., 1994). Thus, permanent and selective deletion of mHTT gene could be an interesting therapeutic approach for HD with no negative effect on patient health. The CRISPR/Cas9 system has been investigated for its utility for HD therapy but is still in the preclinical stage. Using fibroblasts (Shin et al., 2016; Monteys et al., 2017; Dabrowska et al., 2018) and iPSCs-derived NPCs (Shin et al., 2016) from an HD patient, it has been demonstrated that the selective deletion of a fragment of the mHTT gene using the CRISPR/Cas9 strategy is possible. This approach led to a near-complete reduction of mHTT protein and left intact the wild type HTT gene (Shin et al., 2016). Furthermore, using CRISPR/Cas9 and a transposon-based approach, the precise correction of the CAG expansion in HD iPSCs has been achieved (Xu et al., 2017). These cells retain pluripotency, have a normal karyotype, and could be differentiated into NPCs and MSNs-like neurons, which presented some electrical properties of neurons. Interestingly, the corrected cells were deprived of some phenotypic abnormalities observed in the HD iPSC-derived neurons, including increased susceptibility to growth factor withdrawal, impaired neural rosette formation, and mitochondrial dysfunction (Xu et al., 2017).

Using BACHD mice (Table 4) it has been shown that mHTT expression is reduced when Cas9 and a single guide RNA (sgRNA) are delivered using AAVs in the striatum, causing the deletion of a fragment around exon 1 (Monteys et al., 2017). Likewise, AAV-mediated injection of Cas9 and 2 sgRNA into the striatum of HD140Q-KI mice (Table 4) achieved a significant reduction in mHTT levels, accompanied by improved motor performance, decrease in reactive astrocytes and attenuation of bodyweight reduction (Yang et al., 2017). Similar results have been found using the R6/2 mice model, in which AAV-mediated delivery of Cas9 and sgRNA into the striatum caused a near 40% reduction in mHTT inclusions and around a 50% decrease in mHTT protein levels (Ekman et al., 2019). These mice presented increased mean survival, better motor performance, and decreased hindlimb clasping, an established indicator of dystonia (Ekman et al., 2019).

Despite the promising results, there is still a need for validation of this approach for human research. Given that many studies use CRISPR/Cas9 technology based in the recognition of short repeat sequences in the DNA, intensive studies must be performed to find these short sequences in the mHTT gene that is not present in another codifying gene sequence, so off-target genomic removal could be avoided. Moreover, single-nucleotide changes can occur in the mHTT gene in every patient affected, therefore sequencing of the mutant gene would be necessary for each patient. Another important issue of using CRISPR/Cas9 technology in humans is that the deletion of the mutated gene is permanent and irreversible. Also, the transduction of Cas9, a protein of bacterial origin, could activate the patients’ immune system and edited cells would be effectively eliminated (Wignakumar and Fairchild, 2019). Despite this, CRISPR/Cas9-mediated deletion of the mHTT gene could be perfectly coupled with cell replacement strategies, with a correction of the mutation.

A second strategy for lowering mHTT levels is based on RNA-targeting strategies. One of the most studied methods to achieve this is through RNAi. These include the use of microRNAs (miRNAs) and short hairpin RNAs (shRNAs), all using the Dicer-RISC machinery for the targeted degradation of mRNAs (Ghosh and Tabrizi, 2017). Initially, using shRNAs and HD-N171-82Q HD mice (Table 4), it was shown a partial reduction of mHTT mRNA by 50% and decreased mHTT protein accumulation in the striatum, accompanied by a significant improvement in motor performance (Harper et al., 2005). Similarly, using R6/1 HD mice (Table 4), it has been demonstrated that AAV-mediated delivery of shRNAs against mHTT significantly reduces mHTT mRNA and protein levels (Rodriguez-Lebron et al., 2005). shRNA-injected striatum had smaller and less intense intranuclear mHTT inclusions, which was accompanied by increased MSNs mRNA markers. However, this group showed just a mild effect on the clasping phenotype observed in these mice (Rodriguez-Lebron et al., 2005).

These and other studies have demonstrated that the reduction of mHTT using RNAi in the brain of HD mice decreases both mHTT mRNA and protein levels, which is accompanied by improved motor behavior. However, in these studies the RNAi was designed to target human mHTT, leaving endogenous mouse HTT unaltered. This represents a problem when we think about patients’ treatment, given that both alleles differ in the expanded polyQ in the mutated gene and different single nucleotide polymorphisms (SNPs) present. Even though several SNPs have been identified to be differentially present in the mutant and wild type allele, this accounts just for 80% of the population with HD, leaving an important number of patients without a therapeutic alternative (Pfister et al., 2009). Therefore, RNAi has been tested for the reduction of both mHTT and wild type HTT mRNA. Using an HD mouse model that expresses both mouse wild type and human mutant HTT, non-allele specific miRNA-mediated knockdown of both mRNAs in the striatum can significantly improve motor coordination (Boudreau et al., 2009). Notably, these mice survived and had no phenotypic changes after a 75% reduction of wild type HTT for 4 months (Boudreau et al., 2009). Using AAV-mediated miRNA injection into the putamen of rhesus monkeys to target both HTT and mHTT mRNAs, it has been demonstrated that a 45% reduction of HTT mRNA did not induce motor deficits in these animals, nor neurodegeneration, astrogliosis, microglial activation or neuroinflammation (McBride et al., 2011). These phenotypes were also maintained in rhesus monkeys treated with AAV-shRNA for HTT mRNA for 6 months (Grondin et al., 2012).

One important limitation of double-stranded RNAs (shRNAs and miRNAs) is that they are not capable of crossing the BBB and do not easily cross the plasma membrane of cells. Given that they demonstrated positive effects when targeted to the brain, methods to deliver these molecules are under study (Chernikov et al., 2019). Despite this limitation, in 2019 the first clinical trial using AAV5-mediated delivery of a miRNA against HTT was initiated (Table 2, NCT04120493). Early manifest HD patients will be assessed for motor and cognitive functions, with miRNA and exploratory biomarkers measured in the CSF. The same AAV5-miRNA was proven to lower mHTT mRNA specifically in rat and minipig HD model. Suppression of mHTT mRNA levels was associated with lower mHTT protein levels, inclusion formation, and neuronal dysfunction (Miniarikova et al., 2017; Evers et al., 2018).

Another strategy used for RNA targeting is through ASOs. These are short, synthetic single-stranded DNA molecules that are complementary to a pre-mRNA target sequence in the nucleus, and have been widely studied for the treatment of neurological disorders (Rinaldi and Wood, 2018). Using IONIS-HTTRx against HTT mRNA, Tabrizi et al. (2019) reported the first results obtained from a phase I–II multicenter clinical trial in which this ASO was intrathecally administered in early-manifest HD patients. IONIS-HTTRx demonstrated to be safe and well-tolerated, and importantly, mHTT levels in the cerebrospinal fluid (CSF) of patients treated with IONIS-HTTRx decreased dose-dependently. In this trial, no changes in motor and cognitive functions were observed between the placebo and the treated group, which can be explained by the slow progression of the disease and the narrow window of time in which these changes were measured (Tabrizi et al., 2019). Some of these patients participated in a 15-month extension study, which ended by October 2019 (Table 2, NCT03342053). The original clinical trial is now under phase III, with more than 900 patients in 101 locations around the world, which is expected to end by September 2022 (Table 2, NCT03761849).

A second and third phase Ib/IIa clinical trials are underway to evaluate single and multiple doses of two ASOs against two specific SNPs found in the mHTT gene. PRECISION-HD1 clinical trial will evaluate the ASO WVE-120101 against the SNP rs362307 (Table 2, NCT03225833) and PRECISION-HD2 clinical trial will use the ASO WVE-120102 against the SNP rs362331 (Table 2, NCT03225846). These multicenter clinical trials, in which 60 patients with early-manifest HD are participating, started in 2017 and are expected to finish by the end of this year.

The results of the IONIS-HTTRx clinical trial are encouraging and bring us closer to therapy for HD. Given that ASOs bind directly to the DNA sequence, it is less likely that off-target suppression occurs. Moreover, as in the PRECISION clinical trials, unique SNPs found specifically in the mHTT DNA sequence allows for the design of specific ASOs, avoiding off-target suppression. However, the use of ASOs has disadvantages. In the PRECISION clinical trials, the ASOs evaluated target specific SNPs found in the mHTT gene, but they are not present in all HD patients. On the other hand, the IONIS-HTTRx clinical trial targets both wild type and mHTT genes, so it can be used in all HD patients. However, to date, there is no data available on the possible effects of wild type HTT suppression in patients. As mentioned before, non-human primates with sustained suppression of wild type HTT did not show adverse effects in motor and cognitive skills (McBride et al., 2011; Grondin et al., 2012), results that could be escalated to humans. Nevertheless, evaluation of long-term suppression of wild type HTT is key in HD patients. Also, repeated administration of ASOs could be necessary to maintain therapeutic benefits.

## Discussion

PD and HD are movement disorders characterized by the presence of aberrant and unwanted involuntary movements. The main cause for the variety of motor symptoms observed in patients is the selective neuronal loss in brain areas implicated in movement fine-tuning. Despite this knowledge, current treatments are not able to stop, reverse, or slow down PD and HD. Current treatments are directed to keep to line the motor symptoms, but with limited efficacy. Thus, continuous strong efforts are being made for the development of new therapeutic strategies for both diseases. Moreover, the development of small molecules for the treatment of these neurodegenerative diseases has a particularly high failure rate. Thus, strong efforts are being made for the development of new therapeutic strategies for both diseases.

Currently, no approved drugs can modify the progression of either PD or HD. Nevertheless, pharmacology provided therapies to reestablish DA levels in PD patients and to reduce DA neurotransmission in HD patients. Unfortunately, different and aggressive side effects appear during these treatments. New formulations have been developed to control the additional psychomotor and autonomic complications produced by some anti-parkinsonians drugs, however, it is important to reduce all the events that can affect or deteriorate the quality life of patients, and maybe the answers are out of the classical pharmacology approach.

Among the first strategies used for the treatment of PD and HD were cellular replacement therapies, in which fetal tissue was transplanted into the patient’s brain. Even though some functional recovery was observed, this strategy had several limitations. The number of fetuses used generates important religious and ethical concerns. Besides, in some cases, tumor formation was observed in the site of transplantation, and most of the studies required immunosuppressive therapy. Although new strategies have been developed for obtaining dopaminergic and GABAergic neurons from ESCs and iPSCs, these have not yet escalated into clinical trials.

Deregulation of NTFs’ levels has been reported in PD and HD patients, and the administration of NTFs has shown beneficial effects in patients with these diseases. The reported neuroprotective effect can be explained by the prevention of neurodegeneration, restoring, and delaying the occurrence of motor symptoms. Moreover, therapy with NTFs has proven to be effective not only in preclinical stages but has also shown a positive effect in clinical trials. However, one main limitation of NTFs therapy is its poor brain availability after oral administration, which can only be solved by direct administration to the brain. Clinical studies directly administrating NTFs to the brain have reported side effects such as nausea, vomiting, headache, as well as infection when catheter implantation is used. Despite side effects, these studies have observed promising effects with this therapy, however, improvement and development of new delivery methods are key for successful NTF-base treatments.

Without a doubt, the development of electrical neuromodulatory therapies to treat motor symptoms in PD and HD has brought encouraging news for patients, doctors, and their families. Clinical reports carried out in recent decades have validated the use of DBS or SCS as a complement to classical pharmacological therapies that, due to their chronic use, decrease their effectiveness or induce more complex motor symptoms. Additionally, studies in PD patients have reported that the simultaneous use of DBS and SCS would bring important benefits, since DBS is effective in relieving motor symptoms such as tremor, bradykinesia, and stiffness, while SCS has to relieve symptoms associated with walking and posture. Although this has not been validated in the clinic, it opens the discussion about the possibility of using both strategies for the synergistic treatment of cardinal and axial symptoms of PD. When it comes to HD treatment, DBS has emerged as a promising alternative after showing positive effects similar to pallidotomy, reducing choreic movements and dystonia, but with the advantage of being a flexible option that adjusts to the patient’s requirements. Since the mechanisms by which both strategies exert their effect have not been fully elucidated, their use requires personalized patient-to-patient care throughout the treatment, complicating its application in countries with weak health systems. Moreover, given their surgical complexity and high monetary cost, these therapies continue to be considered as a last option, even when reports in pre-clinical models, particularly in the case of PD, have reported encouraging results regarding their effect in counteracting neurodegeneration processes.

Worldwide excitement among HD patients and their families arose after the report of a promising potential therapy (IONIS-HTTR_x_) that was able to decrease mHTT levels in the CSF of patients. Now, this treatment is under phase III clinical trial, in which patients of more than 100 centers around the world have been recruited. This is by far the closest that we have got to a definite treatment for HD. Nevertheless, many questions need to be answered during this clinical trial to ensure safety for patients. Given that IONIS-HTTR_x_ targets both HTT and mHTT, one important question is whether long-term suppression of HTT is deleterious for neurons and if patients present improvements in motor and cognitive function.

Along with the development of new therapeutic approaches, like the ones described in this review, comes the important need to assess therapy response and disease progression. A key line of investigation is focused on finding new and reliable biomarkers that allow assessing changes in the disease course, helping in the evaluation of therapeutic response. Several biomarkers have been described for both PD and HD patients, but global multicenter studies are necessary to validate those biomarkers worldwide. Another important aspect to be considered in the development of new therapies is the heterogeneity of symptoms observed in both PD and HD patients, which suggest that not all treatments are the best option for all cases, as well as not all treatments are the best options for the same symptoms, suggesting that future clinical approaches will personalize the treatment or combine the strategies presented in this article to increase their efficacy and/or reduce its adverse effects.

Altogether, the presented data highlights the idea that we are on the right track for the development of new therapeutic strategies for PD and HD, but there is still a long road ahead to find a definitive treatment that may stop the progression or even cure the disease.

## Authors Contributions

PT-E, DS, RP-A, AP, JA, and RV wrote and edited the manuscript. RV and FG critically analyzed the data. PT-E, AP, JA, and RV prepared the figures and tables. RV planned the manuscript. All authors contributed to the article and approved the submitted version.

## Conflict of Interest

The authors declare that the research was conducted in the absence of any commercial or financial relationships that could be construed as a potential conflict of interest.
